# Adherence and related cardiovascular outcomes to single pill vs. separate pill administration of antihypertensive triple-combination therapy

**DOI:** 10.1097/HJH.0000000000003497

**Published:** 2023-07-05

**Authors:** Federico Rea, Gabriella Morabito, Laura Savaré, Atul Pathak, Giovanni Corrao, Giuseppe Mancia

**Affiliations:** a National Centre for Healthcare Research and Pharmacoepidemiology; b Department of Statistics and Quantitative Methods, University of Milano-Bicocca; c MOX – Laboratory for Modeling and Scientific Computing, Department of Mathematics, Politecnico di Milano; d CHDS – Center for Health Data Science, Human Technopole, Milan; e Department of Cardiology, and UMR UT3 CNRS 5288 Hypertension and Heart Failure: Molecular and Clinical Investigations, INI-CRCT F-CRIN, GREAT Networks, Centre Hospitalier Princesse Grace, Monte Carlo, Monaco; f Emeritus Professor of Medicine, University of Milano-Bicocca, Milan; g Policlinico di Monza, Monza, Italy

**Keywords:** adherence, antihypertensive drugs, clinical outcomes, discontinuation, population-based study, single-pill combination

## Abstract

To compare adherence to antihypertensive treatment between patients prescribed a three-drug single-pill combination (SPC) of perindopril/amlodipine/indapamide (P/A/I) vs. the combination of an angiotensin-converting enzyme inhibitor (ACEI), a calcium-channel blocker (CCB), and a diuretic (D) as a two-drug SPC plus a third drug given separately.

**Methods:**

Using the healthcare utilization database of the Lombardy Region (Italy), the 28 210 patients, aged at least 40 years, who were prescribed P/A/I SPC during 2015–2018 were identified and the date of the first prescription was defined as the index date. For each patient prescribed the SPC, a comparator who started ACEI/CCB/D treatment as a two-pill combination was considered. Adherence to the triple combination was assessed over the year after the index date as the proportion of the follow-up days covered by prescription (PDC). Patients who had a PDC >75% were defined as highly adherent to drug therapy. Log-binomial regression models were fitted to estimate the risk ratio of treatment adherence in relation to the drug treatment strategy.

**Results:**

About 59 and 25% of SPC and two-pill combination users showed high adherence, respectively. Compared with patients under a three-drug two-pill combination, those who were treated with the three-drug SPC had a higher propensity to be highly adherent to the triple combination (2.38, 95% confidence interval: 2.32–2.44). This was the case regardless of the sex, age, comorbidities, and number of co-treatments.

**Conclusions:**

In a real-life setting, patients under three-drug SPC exhibited more frequently a high adherence to antihypertensive treatment than those prescribed a three-drug two-pill combination.

## INTRODUCTION

Single-pill combination (SPC) of two or three antihypertensive drugs is recommended by the guidelines of the European Society of Cardiology and the European Society of Hypertension [1,2] because of the evidence that reduction in the prescribed number of antihypertensive drug tablets to be taken every day is accompanied by an increased adherence to treatment [3]. This is regarded as an important goal to pursue because adherence to treatment is low in hypertension [4] and its increase is associated with an improved rate of blood pressure (BP) control [5] and a reduction in the risk of cardiovascular (CV) morbid and fatal events [6,7]. However, while several studies support the conclusion that SPC of two BP-lowering drugs leads to better adherence to treatment than separate drug administration [8], evidence that this is the case also for SPC of three drugs is more limited and not entirely consistent. While an increased adherence to treatment has been observed in some studies (including a randomized trial of only a few weeks duration) [9,10], other studies have not found any increase in treatment adherence among three-drug SPC users in comparison with separate drug administration of the same drugs, e.g. two-drug SPC plus a third agent given separately [11,12].

The present study had as the primary aim to compare adherence to three-drug antihypertensive treatment in patients prescribed SPC vs. those using a two-pill combination, i.e. a SPC of two antihypertensive agents plus a third antihypertensive agent given separately. A first secondary aim was to also compare the relationship of adherence and clinical outcomes as measured by hospitalization for CV events in the two treatment groups to see whether and to what extent differences in adherence translated into clinical benefits. Comparison between the two treatment strategies was also extended to the overall costs of CV health services, which was determined by cost of drugs, outpatient medical services, and hospitalizations.

## METHODS

### Setting

The data used in the present study were retrieved from the Healthcare Utilization Databases of Lombardy, a Region of Italy that accounts for almost 16% of its population (about 10 million of residents). All Italian citizens have equal access to the healthcare services provided by the National Health Service (NHS) and both in Lombardy and in other regions the related data are included in an automated system of databases which provides information on all health services free of charge, including prescriptions of drugs [classified according to the Anatomical Therapeutic Chemical, (ATC) classification system] to outpatients made by general practitioners or specialists (e.g. cardiologists, internists, etc.), hospitalizations [with diagnoses and procedures coded according to the International Classification of Diseases, 9th Revision, Clinical Modification (ICD-9-CM) classification system], and other healthcare-related items. These databases are linked by a single individual identification code, which allows to trace the healthcare pathway of NHS beneficiaries. To preserve privacy, each identification code is automatically deidentified, the inverse process being only allowed to the Regional Health Authority upon request from judicial authorities. Further details on Healthcare Utilization Databases in our studies and in pharmacoepidemiological studies are available in previous publications [6,13,14].

### Cohort selection and follow-up

The target population included Lombardy residents, aged 40 years or older, who were beneficiaries of the NHS. Of these, those who received a prescription of a SPC of three BP-lowering drugs between 2015 and 2018 were identified and included in the *exposed cohort*. The date of the first prescription during this period was defined as the *index date*. During the study period, only the perindopril/amlodipine/indapamide SPC was available in the Italian market. Patients were excluded if they (i) were not beneficiaries of the NHS for at least 3 years before the index date, (ii) had less than the planned 1 year of follow-up (see below), because of death or change of residence from Lombardy to other regions, and (iii) patients who had only the initial index date prescription, i.e. in whom need for antihypertensive drug treatment was not confirmed.

For each patient included in the exposed cohort, a comparator was identified among subjects prescribed a combination of an angiotensin-converting enzyme inhibitor (ACEI), a calcium-channel blocker (CCB), and a diuretic as a two-drug SPC plus a third drug given separately (i.e. 2 pills). The ACEI, CCB and the diuretic were other than perindopril, amlodipine and indapamide in 85, 52, and 99% of the patients, respectively. Patients using triple therapy in a two-drug combination were selected provided that (i) the above-mentioned inclusion and exclusion criteria were satisfied and (ii) the index date fell within ±30 days of the index date of the corresponding three-drug SPC patient. The identified comparators were included in the *nonexposed cohort*.

Patients prescribed the three-drug SPC and the corresponding comparators had also to be prescribed a similar antihypertensive treatment strategy during the year before the index date. The cohort members were followed from the index date for 365 days afterwards.

### Adherence to and discontinuation of antihypertensive drug therapy

For each patient included in the exposed and nonexposed cohorts, all antihypertensive drugs dispensed during the follow-up were identified. Because in our database the prescribed daily doses are not recorded, the period covered by a prescription was calculated by dividing the total amount of the drug prescribed for the defined daily dose. For overlapping prescriptions, the patient was assumed to have taken all the drug(s) contained in the former prescription before starting the latter one. Adherence to antihypertensive drug therapy was assessed by the ratio between the number of days in which the triple combination was available and the days of follow-up, a measure defined as ‘proportion of days covered’ (PDC) by prescriptions [15]. Because information on drug therapies dispensed during hospitalizations was not available, the triple combination was assumed to be prescribed during a hospitalization period [16].

The primary goal of the study was to compare the odds of being highly adherent to the triple combination treatment (PDC > 75%) between the above-mentioned two groups. Secondary goals were to compare the odds of (i) being poorly adherent to treatment (PDC < 25%), and (ii) treatment discontinuation. These cut-off values were used because in previous studies on the Lombardy database these adherence levels showed a clear association with a reduction and an increase of CV outcomes and mortality, respectively [6,17]. Treatment discontinuation was assumed if the time-span between the end of one prescription and the beginning of the following one was greater than 90 days [15]. Among patients prescribed a two-drug SPC combination plus a third drug separately, treatment discontinuation was assumed if at least one pill was discontinued.

### Covariates

Baseline characteristics included sex, age, use of other drugs (statins, antidiabetic drugs, etc.), and previous hospitalization for CV events (heart failure, myocardial infarction, and stroke). In addition, the number of co-medications dispensed in the year prior to the index date was assessed and categorized as 0–4, 5–9, and ≥10. Finally, the clinical profile was assessed by the Multisource Comorbidity Score, a prognostic score that has been shown to predict all-cause death of Italian people more accurately than other widely used scores [18]. Three categories of clinical profile were considered: good (0 ≤ score ≤ 4), intermediate (5 ≤ score ≤ 14), and poor (score ≥ 15).

### Clinical outcomes

The clinical outcome of interest was hospitalization for major CV events, that is stroke, myocardial infarction and/or heart failure listed as the primary diagnosis. The clinical outcomes were assessed over the follow-up period after the evaluation of drug adherence, that is, from 1 year after the index date until censoring (the earliest among emigration, death, or data availability, that is, December 31, 2019).

### Healthcare costs

Healthcare costs were calculated by the amount that the Regional Health Authority reimbursed to health providers. Costs included hospitalization for CV diseases, antihypertensive drugs, and outpatient services for CV care (specialist visits, laboratory examinations, imaging, etc.). Healthcare costs were assessed for the year after the index date.

### Data analysis

To address the main goal of the study, patients were classified according to the treatment strategy, that is, whether the three antihypertensive drugs were prescribed as a SPC or as a two-pill combination, according to the intention-to-treat approach.

The standardized mean differences were used to compare differences between groups [19]. Standardized mean differences <0.10 were considered negligible.

Log-binomial regression models were fitted to estimate the risk ratio (RR), and its 95% confidence interval (CI), of treatment adherence and discontinuation in relation to drug strategy, using the group taking a SPC of two drugs plus a third drug separately as reference. Adjustments were made for the baseline covariates. The analyses were repeated after stratification of patients for sex, age, number of co-medications, and clinical status.

To address the main second goal of the study, that is, whether and to what extent adherence was related to clinical outcomes and the relationship was different in the two treatment strategy groups the following analyses were performed. First, two Cox models were fitted to estimate the hazard ratio (HR), and its 95% confidence interval (CI), of drug adherence on clinical outcomes after adjusting for baseline covariates and stratifying the data for the treatment strategy. Second, we summarized estimates by means of meta-analytic procedures. Heterogeneity between treatment strategies was tested by Cochran's *Q* test and measured with the *I*^2^ statistic [20].

A linear regression model was fitted to compare the mean of healthcare costs between groups, adjusting for the above-mentioned covariates.

### Sensitivity analyses

To verify the robustness of our findings, four further analyses were performed. First, because of the arbitrary nature of the PDC categorization, we used more permissive and restrictive PDC categories (70 and 80% of the follow-up time, respectively) to define high adherence to treatment. Second, the association between treatment strategy and drug adherence was compared over a longer time window, that is, a follow-up of 2 years. Third, to match the clinical characteristics of the exposed and nonexposed groups, a 1:1 *propensity score matching design* was adopted [21]. The propensity to be prescribed the three-drug SPC was derived through a logistic regression model, which included the above-mentioned covariates. For each patient treated with the three-drug SPC, one patient under the three-drug two-pill combination was randomly selected to be matched for propensity score using a nearest-neighbor matching algorithm without replacement [22]. Fourth, we increased the therapeutic coverage of each prescription up to two times in patients who received antihypertensive drug treatment as a single pill of two drugs plus a third drug given separately to account for the possibility that in these patients daily drug doses were smaller than the defined ones, with thus a greater duration of the prescription and an underestimation of adherence in the group compared with the SPC one.

The Statistical Analysis System Software (version 9.4; SAS Institute, Cary, North Carolina, USA) was used for the analyses. For all hypotheses tested, two-tailed *P* values <0.05 were considered to be significant.

## RESULTS

### Patients

Among patients who SPC of three antihypertensive drugs was prescribed, 30 617 individuals met the inclusion criteria. Of these, 28 210 patients were matched to 28 210 subjects prescribed a two-drug SPC combination plus a third drug separately. As shown in Table S1, Supplemental Digital Content, patients prescribed the three-drug SPC and those prescribed the three drugs as two-pill combination had superimposable antihypertensive treatment strategies during the year before the index date. During this period, about two-thirds of the patients were prescribed three BP-lowering drugs and more than one patient out of five added a third antihypertensive agent to a previous two-drug combination.

The baseline characteristics of the included patients are shown in Table 1. Compared with patients on the three-drug two-pill combination, those treated with a three-drug SPC were slightly younger and more frequently males. There were no other between-group differences in the covariates distribution.

**TABLE 1:** Characteristics of patients prescribed the single-pill combination (SPC) and of the matched patients prescribed the two-pill combination

	Patients on SPC (*N* = 28 210)	Patients on two-pill combination (*N* = 28 210)	Standardized differences
Men	15 231 (54.0%)	13 446 (47.7%)	0.127
Age (years), mean [SD]	67.8 [11.6]	70.9 [11.8]	0.254
Other drugs			
Statins	11 042 (39.1%)	11 151 (39.5%)	0.008
Antidiabetic drugs	6223 (22.1%)	5538 (19.6%)	0.060
Number of co-treatments			0.037
0–4	15 925 (56.5%)	15 739 (55.8%)	
5–9	9636 (33.2%)	9793 (39.7%)	
≥10	2649 (9.4%)	2649 (9.5%)	
Previous hospitalizations			
Stroke	899 (3.2%)	1051 (3.7%)	0.030
Heart failure	426 (1.5%)	559 (2.0%)	0.036
Myocardial infarction	469 (1.7%)	577 (2.1%)	0.028
Clinical profile^a^			0.071
Good	19 479 (69.0%)	18 743 (66.4%)	
Intermediate	8199 (29.1%)	7624 (27.0%)	
Poor	1268 (4.5%)	1112 (3.9%)	

a Three categories were considered for the clinical profile according to the Multisource Comorbidity Score (MCS): good (0 ≤ MCS ≤ 4), intermediate (5 ≤ MCS ≤ 14), and poor (MCS ≥ 15).

### Adherence to and discontinuation of the triple drug therapy

**FIGURE 1:**
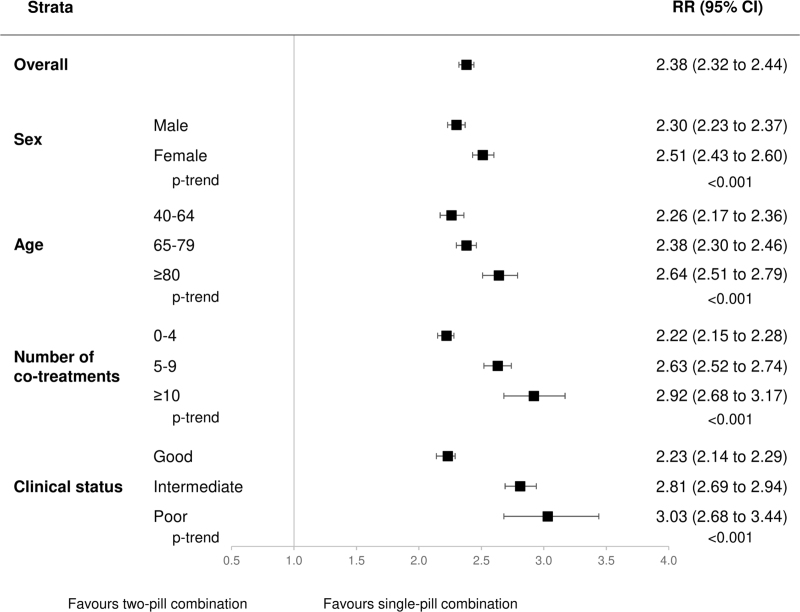
Risk ratios (RR), and 95% confidence intervals (CI), estimating the association between high adherence to treatment (PDC > 75%) and single-pill combination vs. the two-pill combination. Three categories were considered for the clinical profile according to the Multisource Comorbidity Score (MCS): good (0 ≤ MCS ≤ 4), intermediate (5 ≤ MCS ≤ 14), and poor (MCS ≥ 15).

High adherence to treatment (PDC > 75%) was observed in 59% of patients under three-drug SPC therapy and in 25% of those under three-drug two-pill combination. As shown in Fig. 1, compared to those prescribed three drugs as a two-pill combination, patients on three-drug SPC had a greater chance of being highly adherent to treatment (RR = 2.38, 95% CI: 2.32–2.44, *P* < 0.001). This was the case for all strata of age, sex, clinical status, and number of co-medications. The benefit was greater among women, elderly patients, patients under polypharmacy, and those with poor clinical status (*P*-trend < 0.001).

**FIGURE 2:**
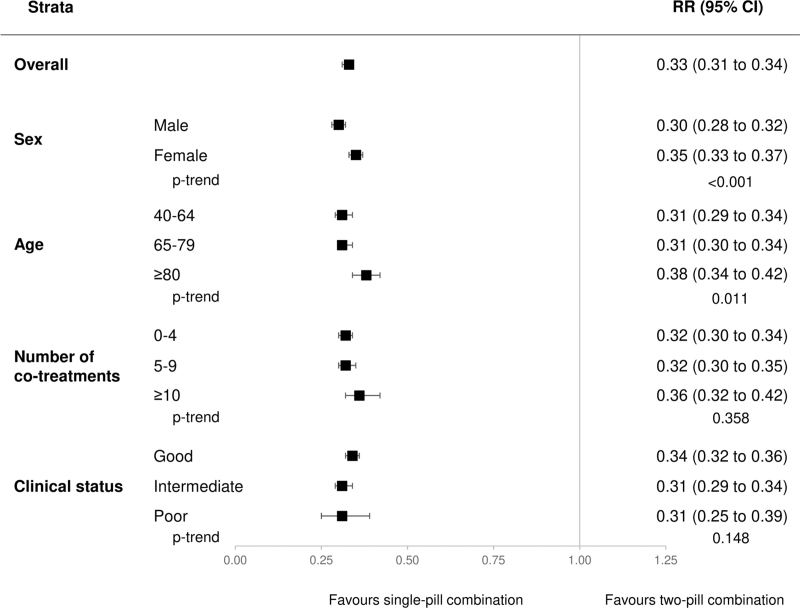
Risk ratios (RR), and 95% confidence intervals (CI), estimating the association between low adherence to treatment (PDC < 25%) and single-pill combination vs. the two-pill combination. Three categories were considered for the clinical profile according to the Multisource Comorbidity Score (MCS): good (0 ≤ MCS ≤ 4), intermediate (5 ≤ MCS ≤ 14), and poor (MCS ≥ 15).

Conversely, the risk of being low adherent to treatment (PDC < 25%) involved eight and 23% of the patients taking the three antihypertensive drugs as SPC and two-pill combination, respectively. As shown in Fig. 2, the three-drug SPC reduced by more than two-thirds the propensity of being low adherent (67%, 66–69%, *P* < 0.001). The effect was similarly marked regardless of clinical status and the number of comedications, but greater among men and younger patients.

**FIGURE 3:**
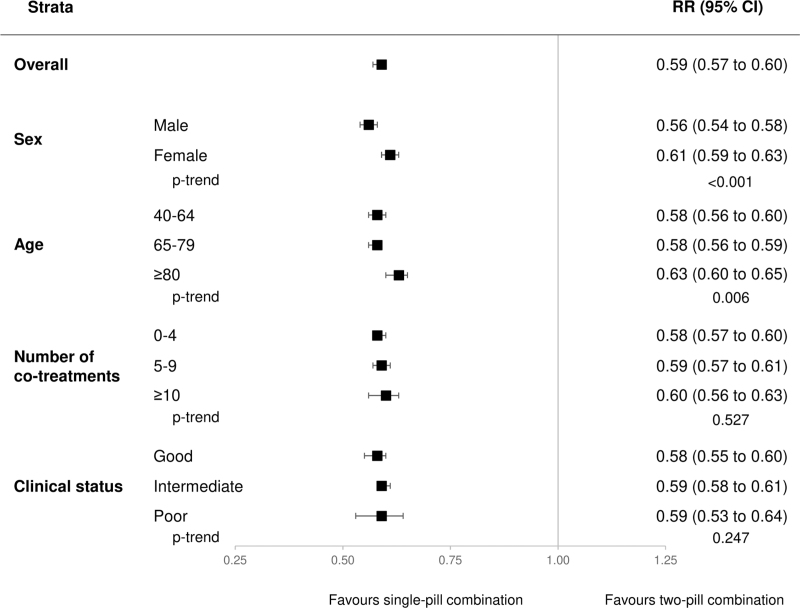
Risk ratios (RR), and 95% confidence intervals (CI), estimating the association between treatment discontinuation and single-pill combination vs. the two-pill combination. Three categories were considered for the clinical profile according to the Multisource Comorbidity Score (MCS): good (0 ≤ MCS≤4), intermediate (5 ≤ MCS ≤ 14), and poor (MCS ≥ 15).

About four of 10 patients discontinued the triple combination, 31% among the three-drug SPC users and 53% among the three-drug two-pill combination users. As shown in Fig. 3, compared to the three-drug two-pill combination, patients under three-drug SPC had a much lower risk of treatment discontinuation (41%, 40–43%, *P* < 0.001). The effect was more pronounced among men and younger patients, while it was similar regardless of the clinical status and number of co-medications.

### Adherence to antihypertensive treatment and clinical outcomes

The cohort members accumulated 105 465 person-years of observation (on average, 1.9 years per patient) and generated 2441 CV hospitalizations. There were 207 outcomes every 10 000 person-years among patients on SPC, and 246 outcomes every 10 000 person-years among those under the three drug two-pill combination.

**FIGURE 4:**
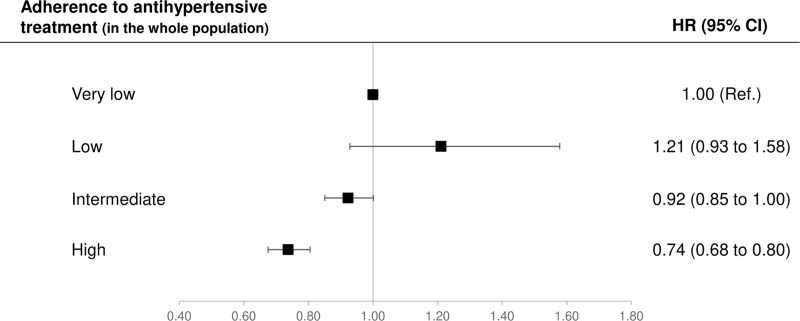
Hazard ratios (HR), and 95% confidence intervals (CI), for cardiovascular hospitalization associated with adherence to antihypertensive drugs, after adjustment for the baseline characteristics. Meta-analytic procedures were used for summarized estimates. Adherence categories are: very low (PDC < 25%), low (25% ≤ PDC < 50%), intermediate (50 ≤ PDC ≤ 75%), and high (PDC >75).

According to the summarized estimates, in the whole study population there was a progressive reduction in the adjusted risk of hospitalization as adherence increased from very low to high levels (Fig. 4). Compared with very low adherence, patients with intermediate and high adherence showed an adjusted risk reduction of 8% (0–15%) and 26% (20–32%), respectively. There was no evidence that these risk reductions differed between treatment strategy groups (*P*-value = 0.066 and 0.923, respectively) (Figure S1, Supplemental Digital Content).

### Healthcare costs

The mean healthcare cost was € 721 among patients under three-drug SPC and €811 under three-drug two-pill combination (Table S2, Supplemental Digital Content). According to the linear model, three-drug SPC users had €64 lower costs for CV healthcare services (*P* < 0.001). The difference was largely driven by a reduction in hospitalization costs (€59), whereas the costs of drugs and outpatient services were similar between the two groups.

### Sensitivity analyses

As shown in Tables S3–S5, Supplemental Digital Content, the results described in the previous sections did not change by modifying the PDC categorization, extending the follow-up during which adherence was measured to two years, or by adopting the propensity score matching design. As shown in Figure S2, Supplemental Digital Content, patients on three-drug two-pill combination should have halved the daily dose to nullify the observed between-group difference in treatment adherence.

## DISCUSSION

Our study provides a number of novel findings. First, the chance of being highly adherent to the triple combination of antihypertensive drugs was significantly greater in patients on the three-drug SPC than in those prescribed the same three drug classes in a two-pill combination. Second, compared to the three-drug two-pill combination, the three-drug SPC strategy reduced the risk of patients to be poorly adherent to the treatment regimen as well as to discontinue the three prescribed drugs. Third, the benefits of the three-drug SPC strategy were shared by males and females, younger and older patients, patients with different CV risk levels and patients with widely different daily pill loads. Fourth, the advantages of assuming three drugs as SPC were by no means quantitatively marginal because (i) in the entire cohort this conferred a 138% increase in the propensity to be highly adherent, while reducing the risk of being poorly adherent by 67%, and (ii) these benefits were rather uniform in patients with different demographic and clinical characteristics. Thus, taking three BP-lowering drugs as SPC leads to a substantial and widely distributed improvement in adherence to antihypertensive treatment compared to the assumption of three drugs as SPC of two drugs plus a separate addition of a third drug as it may often happen in clinical practice.

Other findings of our study deserve to be mentioned. One, three-drug SPC improved treatment adherence and reduced treatment discontinuation also when follow-up was extended to 2 years. This suggests that the improvement of adherence is not temporary but long-lasting, which is of basic importance for a chronic treatment such as hypertension. Two, improvement of adherence translates into an important clinical advantage because adherence is inversely related to the risk of CV outcomes and mortality in a variety of studies [6,7,23–25], which have also shown a sizeable increase in CV risk after treatment discontinuation [26]. Our study confirms these findings because in our patients the higher was adherence, the lower was the risk of hospitalization for CV causes. In this context, however, a further interesting finding is that there was no difference in the risk reduction associated with drug adherence between the three-drug SPC and the two-pill combination, that is, two drugs as a SPC and the third drug administered separately. This suggests that increase of adherence to antihypertensive drug therapy is beneficial regardless of the treatment strategy and the differences in the individual drugs used (perindopril, indapamide, and amlodipine vs. other ACEIs, CCBs, and thiazide diuretics). Three, compared to the three-drug two-pill strategy, the SPC strategy was also associated with lower healthcare costs, largely because the reduced admissions to hospital care associated with use of three-drug SPC had a marked cost-saving effect. Finally, our study confirms that adherence to antihypertensive treatment is low in medical practice and that this involves also treatments based on multiple BP-lowering drug combinations.

Our study has several elements of strength. First, the investigation was based on a large and unselected population, which was made possible because the data involved virtually all citizens [6,13,14]. Second, studying adherence in unaware patients and doctors avoided the adherence bias associated with patient monitoring [27]. Third, the database we used provided accurate data because pharmacists are required to report prescriptions in detail to obtain reimbursement, and incorrect reports have legal consequences [28]. Finally, the robustness of our main findings was confirmed by the sensitivity analyses in which more permissive (70%) and more restrictive (80%) categories of PDC were considered to define high adherence to the drug treatment and by the adoption of the propensity score matching design.

There are also limitations, however. One, misclassification of drug exposure cannot be completely excluded because our data are drawn from drug prescriptions within the NHS reimbursement system. That is, measurement of drug consumption and prescriptions provided by doctors at the private practice level are not included [13]. However, given their free of charge availability, prescriptions of CV drugs outside NHS are known to be only 6% of the total amount [29]. Two, use of the defined daily dose may not always reflect the prescribed daily doses, which is not included in our database. However, according to one sensitivity analysis, only a strong between-group difference in the daily drug dosage would have nullified the favorable effect of three-drug SPC on adherence to treatment. Three, because antihypertensive drugs are also prescribed for coronary heart disease and heart failure, our data might not exclusively reflect adherence to drug treatment in hypertensive patients and our results may be affected by an unbalanced distribution of CV diseases between the two groups. However, antihypertensive treatment accounts by far for the largest use of ACEIs, CCBs and diuretics in Italy [30]. This is even more the case when these drugs are used in a two or a three-pill combination. Four, only the perindopril/amlodipine/indapamide SPC was available in the Italian market during the study follow-up (2015–2019). Therefore, our results specifically refer to the effect of this three-drug SPC and whether they reflect what happens with three-drug combinations of different drug classes remains to be studied.

Finally, because the Lombardy administrative database does not include clinical data such as BP, our study does not offer information on whether and to what extent better adherence to treatment in the three-drug SPC cohort translated into lower BP values and more frequent BP control compared to three-drug two-pill combination. This is likely, however, because a relationship between adherence and BP control has been repeatedly reported [31,32]. Furthermore, there is abundant evidence of a relationship between adherence, BP, and CV risk [6,7,23–25,33,34]. In this context, a further strength of our study is that adherence was related with CV events.

In conclusion, the SPC of three antihypertensive drugs substantially improved adherence to drug therapy compared to the combination of the same three antihypertensive drug classes in a two-pill modality, that is, a two-drug combination and a third drug given separately. This is the case regardless of the age, sex, and patients’ background clinical condition. In addition, due to the increase in treatment adherence, SPC reduced the risk of CV outcomes and costs of health services. Therefore, the use of three-drug SPC can improve CV protection in patients who need more than two antihypertensive drugs to achieve BP control and reduce costs for the healthcare system.

## ACKNOWLEDGEMENTS

This study was supported by grants from the Italian Ministry of the Education, University and Research (‘Fondo d’Ateneo per la Ricerca’ portion, year 2020), from the Italian Ministry of Health (‘Ricerca Finalizzata 2016’, NET-2016-02363853), and from Servier (grant number H43C22000370007). The funding sources had no role in the design of the study, the collection, analysis and interpretation of the data, or the decision to approve publication of the finished manuscript.

### Conflicts of interest

Giovanni Corrao received research support from the European Community (EC), the Italian Medicines Agency (AIFA), Italian Ministry of Health, and the Italian Ministry of Education, University and Research (MIUR). He took part to a variety of projects that were funded by pharmaceutical companies (i.e., Novartis, GSK, Roche, AMGEN, BMS and Servier). He also received honoraria as member of Advisory.

Giuseppe Mancia received honoraria for participation as speaker/chairman in national/international meetings from Bayer, Boehringer Ingelheim, CVRx, Daiichi Sankyo, Ferrer, Medtronic, Menarini Int., Merck, Novartis, Recordati and Servier.

Other authors have no disclosures.

## Supplementary Material

Supplemental Digital Content
