# Supplementary material for: Adherence and related cardiovascular outcomes to single pill vs. separate pill administration of antihypertensive triple-combination therapy
Source: J Hypertens. 2023 Jul 5;41(9):1466–73. doi: 10.1097/HJH.0000000000003497 (PMC10399952; doi:10.1097/HJH.0000000000003497)

**Adherence and related cardiovascular outcomes to single pill vs separate pill administration of antihypertensive triple-combination therapy**

Federico REA ^a,b^, Gabriella MORABITO ^a,b^, Laura SAVARÉ ^a,c,d^, Atul PATHAK ^e^, Giovanni CORRAO ^a,b^, Giuseppe MANCIA ^f,g^

^a^ National Centre for Healthcare Research and Pharmacoepidemiology, Milan, Italy

^b^ Department of Statistics and Quantitative Methods, University of Milano-Bicocca, Milan, Italy

^c^ MOX - Laboratory for Modeling and Scientific Computing, Department of Mathematics, Politecnico di Milano, Milan, Italy

^d^ CHDS - Center for Health Data Science, Human Technopole, Milan, Italy

^e^ Department of Cardiology, and UMR UT3 CNRS 5288 Hypertension and heart failure: molecular and clinical investigations, INI-CRCT F-CRIN, GREAT Networks, Centre Hospitalier Princesse Grace, Monte Carlo, Monaco

^f^ Emeritus Professor of Medicine, University of Milano-Bicocca, Milan, Italy

^g^ Policlinico di Monza, Monza, Italy

**SUPPLEMENTARY MATERIAL**

**Supplementary Table S1.** Antihypertensive treatment strategy in the year before the cohort entry of patients on three-drug single-pill combination (SPC) perindopril/amlodipine/indapamide and of the control patients prescribed three drugs from the same classes as a two-pill combination, two drugs as single pill plus a third drug separately

| **Antihypertensive treatment strategy** | **Three-drug SPC**  **(N=28,210)** | **Three-drug**  **two-pill combination**  **(N=28,210)** |
| --- | --- | --- |
| No antihypertensive drugs | 1,088 (3.9%) | 1,088 (3.9%) |
| Monotherapy | 2,124 (7.5%) | 2,124 (7.5%) |
| Dual combination between an ACEI, a CCB and a diuretic | 4,107 (14.6%) | 4,107 (14.6%) |
| Another dual combination | 2,094 (7.4%) | 2,094 (7.4%) |
| Triple combination of ACEI/CCB/diuretic | 5,076 (18.0%) | 5,076 (18.0%) |
| Another triple combination | 13,721 (48.6%) | 13,721 (48.6%) |

ACEI: angiotensin-converting enzyme inhibitor; CCB: calcium-channel blocker

**Supplementary Table S2.** Average healthcare cost (Euro) per patient according to drug treatment strategy (three-drug SPC vs. three-drug two-pill combination)

|  | **Three-drug SPC**  **(N=28,210)** | **Three-drug**  **two-pill combination**  **(N=28,210)** |
| --- | --- | --- |
| Hospitalizations | 393 | 477 |
| Antihypertensive drugs | 261 | 272 |
| Outpatient services | 67 | 62 |
| **Total** | 721 | 811 |

**Supplementary Table S3.** Risk ratios (RR), and 95% confidence intervals (CI), estimating the association between high adherence to treatment and drug treatment strategy (three-drug SPC vs. three-drug two-pill combination), according to different thresholds of drug adherence

| Strata | Adherence: PDC>70% | | Adherence: PDC>80% | |
| --- | --- | --- | --- | --- |
|  | **RR** | **95% CI** | **RR** | **95% CI** |
| Overall | 2.15 | 2.10-2.19 | 2.75 | 2.67-2.82 |
| Sex |  |  |  |  |
| Male | 2.05 | 2.00-2.11 | 2.60 | 2.51-2.70 |
| Female | 2.27 | 2.20-2.33 | 2.93 | 2.82-3.06 |
| Age |  |  |  |  |
| 40-64 | 2.07 | 2.00-2.15 | 2.52 | 2.40-2.67 |
| 65-79 | 2.13 | 2.07-2.19 | 2.75 | 2.65-2.85 |
| ≥80 | 2.33 | 2.22-2.44 | 3.13 | 2.95-3.33 |
| Number of co-treatments |  |  |  |  |
| 0–4 | 2.02 | 2.42-2.59 | 2.51 | 2.42-2.59 |
| 5–9 | 2.31 | 2.23-2.39 | 3.10 | 2.95-3.25 |
| ≥10 | 2.53 | 2.35-2.72 | 3.35 | 3.03-0.70 |
| Clinical profile ^§^ |  |  |  |  |
| Good | 2.03 | 1.98-2.08 | 2.53 | 2.46-2.62 |
| Intermediate | 2.41 | 2.32-2.51 | 3.29 | 3.11-3.47 |
| Poor | 2.73 | 2.45-3.06 | 3.58 | 3.10-4.15 |

^§^ Three categories were considered for the clinical profile according to the Multisource Comorbidity Score (MCS): good (0≤MCS≤4), intermediate (5≤MCS≤14), and poor (MCS≥15).

**Supplementary Table S4.** Risk ratios (RR), and 95% confidence intervals (CI), estimating the association between drug treatment strategy (three-drug single-pill vs. three-drug two-pill combination) and outcomes (high adherence to treatment (PDC>75%), low adherence to treatment (PDC<25%), and treatment discontinuation) over 2 years of follow-up

| Outcome | RR | 95% CI |
| --- | --- | --- |
| High adherence | 2.47 | 2.38-2.56 |
| Low adherence | 0.44 | 0.42-0.47 |
| Discontinuation | 0.72 | 0.70-0.73 |

These analyses involve the 14,463 pairs of patients prescribed the SPC and the two-pill combination with a follow-up of at least 2 years.

**Supplementary Table S5.** Risk ratios (RR), and 95% confidence intervals (CI), estimating the association between high adherence to treatment and drug treatment strategy (three-drug SPC vs. three-drug two-pill combination) adopting the propensity score matching design

| Strata | RR | 95% CI |
| --- | --- | --- |
| Overall | 2.34 | 2.29-2.39 |
| Sex |  |  |
| Male | 2.27 | 2.20-2.33 |
| Female | 2.47 | 2.39-2.56 |
| Age |  |  |
| 40-64 | 2.23 | 2.15-2.31 |
| 65-79 | 2.35 | 2.28-2.43 |
| ≥80 | 2.62 | 2.48-2.77 |
| Number of co-treatments |  |  |
| 0–4 | 2.19 | 2.13-2.26 |
| 5–9 | 2.48 | 2.39-2.58 |
| ≥10 | 3.17 | 2.91-3.46 |
| Clinical profile ^§^ |  |  |
| Good | 2.18 | 2.12-2.24 |
| Intermediate | 2.81 | 2.68-2.94 |
| Poor | 2.98 | 2.63-3.38 |

^§^ Three categories were considered for the clinical profile according to the Multisource Comorbidity Score (MCS): good (0≤MCS≤4), intermediate (5≤MCS≤14), and poor (MCS≥15).

**Supplementary Figure S1.** Treatment strategy-specific and summarized hazard ratios (HR), and 95% confidence intervals (CI), estimating the association between adherence to treatment and hospitalization for cardiovascular events


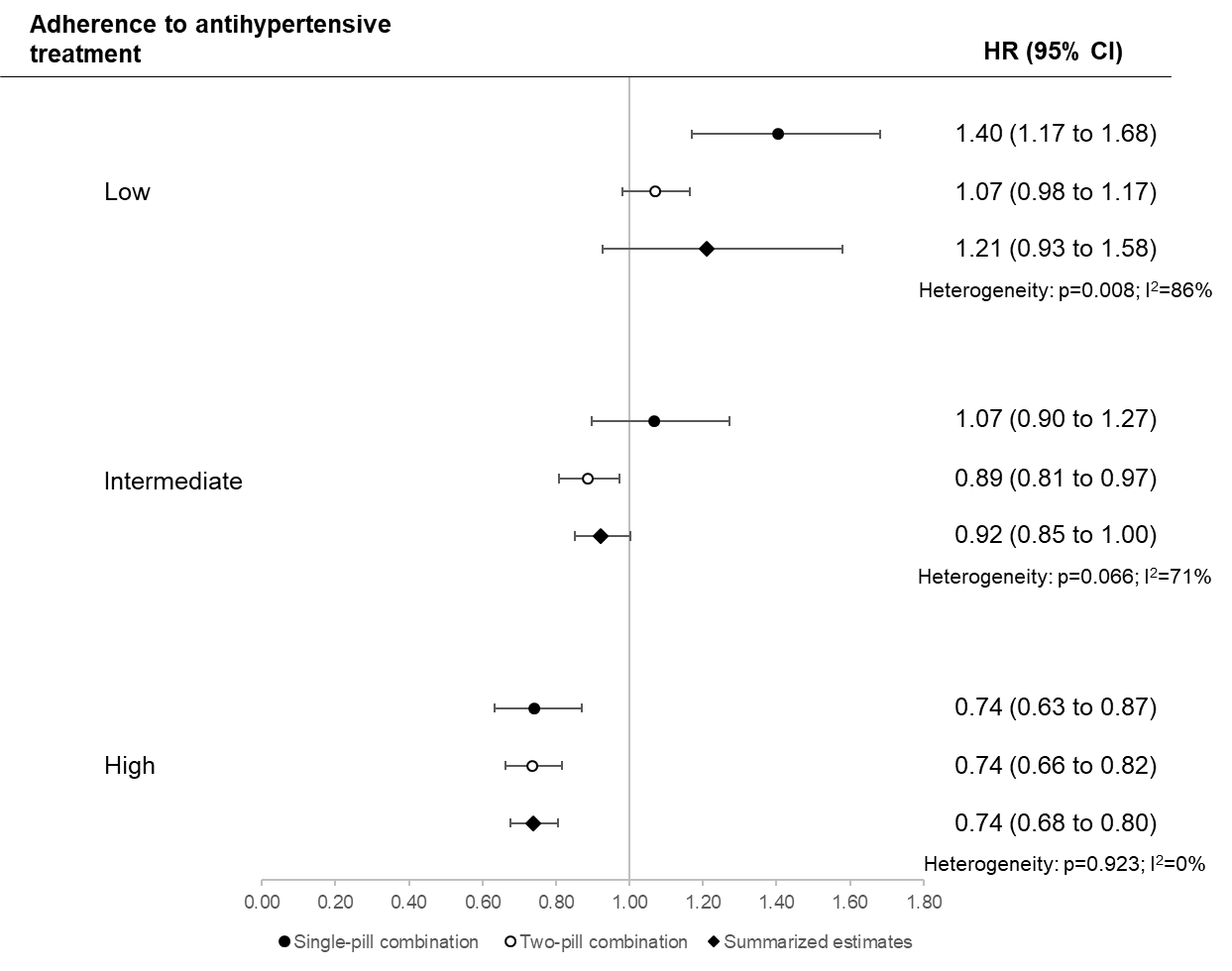


Adherence categories are: very low (PDC <25%), low (25%≤ PDC <50%), intermediate (50≤ PDC ≤75%), and high (PDC >75)

**Supplementary Figure S2.** Risk ratios (RR), and 95% confidence intervals, estimating the association between high adherence to treatment (PDC>75%) and treatment strategy by varying the ratio of daily dose dispensed between patients under three-drug SPC and three-drug two-pill combination


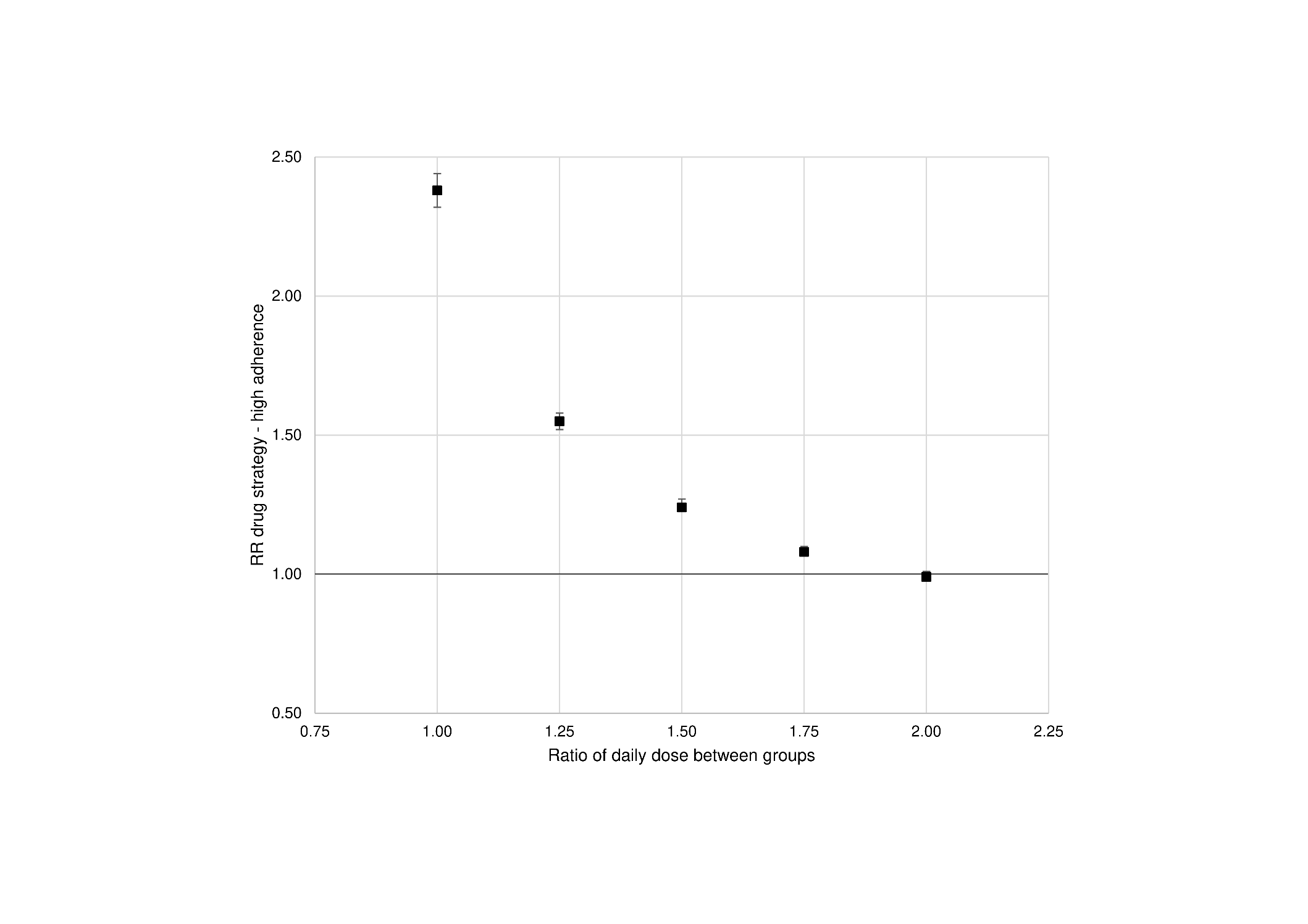

Supplement: Supplemental Digital Content [file jhype-41-1466-s001.docx]
